# The Role of Microglia and Macrophages in CNS Homeostasis, Autoimmunity, and Cancer

**DOI:** 10.1155/2017/5150678

**Published:** 2017-12-19

**Authors:** Jie Yin, Katherine L. Valin, Michael L. Dixon, Jianmei W. Leavenworth

**Affiliations:** ^1^Department of Neurosurgery, University of Alabama at Birmingham, Birmingham, AL 35233, USA; ^2^Department of Microbiology, University of Alabama at Birmingham, Birmingham, AL 35233, USA; ^3^Department of Cell Biology, Tianjin Medical University, Tianjin 300070, China

## Abstract

Macrophages are major cell types of the immune system, and they comprise both tissue-resident populations and circulating monocyte-derived subsets. Here, we discuss microglia, the resident macrophage within the central nervous system (CNS), and CNS-infiltrating macrophages. Under steady state, microglia play important roles in the regulation of CNS homeostasis through the removal of damaged or unnecessary neurons and synapses. In the face of inflammatory or pathological insults, microglia and CNS-infiltrating macrophages not only constitute the first line of defense against pathogens by regulating components of innate immunity, but they also regulate the adaptive arms of immune responses. Dysregulation of these responses contributes to many CNS disorders. In this overview, we summarize the current knowledge regarding the highly diverse and complex function of microglia and macrophages during CNS autoimmunity—multiple sclerosis and cancer—malignant glioma. We emphasize how the crosstalk between natural killer (NK) cells or glioma cells or glioma stem cells and CNS macrophages impacts on the pathological processes. Given the essential role of CNS microglia and macrophages in the regulation of all types of CNS disorders, agents targeting these subsets are currently applied in preclinical and clinical trials. We believe that a better understanding of the biology of these macrophage subsets offers new exciting paths for therapeutic intervention.

## 1. Introduction

The central nervous system (CNS) has been long recognized as an immune-privileged site [1]. But over the last several years, evidence has accrued suggesting that the CNS contains resident immune cells that actively participate in immune surveillance and shape the CNS development and neuronal function under steady states. These resident cells include various types of macrophages, including the most abundant and best studied population, microglia [2]. In the face of pathological insults, CNS microglia and macrophages, including CNS-infiltrating macrophages derived from circulating monocytes, constitute the first line of defense against pathogens by regulating components of both innate and adaptive immune responses. Dysregulation of these responses underlies the pathogenesis of many CNS disorders. Here, we summarize the current understanding of CNS microglia and macrophages, including their development, homeostasis, and functions in physiological and pathological status (autoimmune disease and tumor), the interaction of CNS microglia and macrophages with other immune components (innate and adaptive immune cells), and the therapeutic potential of CNS microglia and macrophages as drug targets.

## 2. The Development, Homeostasis, and Function of CNS Microglia and Macrophages

Macrophages are myeloid cells that survey their immediate and local environment by ingesting and degrading dead cells, debris, and potentially hazardous agents, such as pathogens [3, 4]. As part of the mononuclear phagocyte system, macrophages are present in almost all tissues and have a crucial role in maintaining tissue homeostasis during development and in adulthood. Tissue-resident macrophages are nonmigratory cells that comprise many subsets, including microglia (brain), osteoclasts (bone), alveolar macrophages (lung), histiocytes (interstitial connective tissue), and Kupffer cells (liver). There are also various mononuclear phagocyte subpopulations in the circulation that can differentiate into macrophages once they migrate into tissues, called monocyte-derived macrophages [5, 6]. Although the phenotypes and names of these macrophage populations vary on the basis of their anatomical location, they all acquire similar functional capability when stimulated appropriately [7]. Here, we summarize the current view of the developmental requirement and functional specialization of CNS microglia and macrophages.

### 2.1. The Development and Homeostasis of CNS Microglia and Macrophages

Most tissue-resident macrophages are prenatally established and then maintained through adulthood [8]. Embryonic yolk sac and fetal liver-derived macrophage precursors are the origin of all tissue-resident macrophages, although the contributions of these two progenitors vary among different tissues [8]. Primitive macrophages in the yolk sac appear around embryonic day 7 (E7) and disseminate throughout embryonic tissues following the establishment of blood circulation around E9.5. Fetal liver monocytes infiltrate peripheral tissues, except the CNS, and give rise to tissue-resident macrophages. While macrophages from both origins usually coexist, the fetal liver-derived cells can progressively outcompete yolk sac-derived tissue macrophages. Thus, the generation and maintenance of tissue-resident macrophages are independent from ongoing hematopoiesis, despite the fact that these cells can be complemented by adult monocyte-derived macrophages [9]. For example, during adulthood, bone marrow-derived circulating Ly6C^hi^ monocytes can give rise to relatively short-lived, non-self-renewing tissue-resident macrophages in organs, such as the intestine, heart, and remodeling mammary glands [5, 6]. Despite the similarities of microglia with various other tissue-resident macrophages, two remarkable properties of microglia are their restricted prenatal origin and their capacity for self-renewal and longevity. After birth, myeloid progenitors from the circulation cannot significantly contribute to the pool of adult microglia, and the increase in microglial cell number results from the expansion of resident microglia [10, 11]. While the numbers of microglia increase during aging, their structure changes from a highly ramified shape to a morphology with less elaborate processes accompanied by an irregular tissue distribution pattern and slower responses to environmental signals [12, 13]. In contrast to microglia, circulating monocytes and other tissue macrophages are continually replaced by circulating myeloid cells after birth [14].

In the steady state, the CNS hosts several myeloid populations, including parenchymal microglia, perivascular cells, meningeal macrophages, and choroid plexus macrophages [15]. CNS macrophages have been characterized and classified mainly according to their localization, morphology and surface-marker expression, and *in vitro* responses. Despite the fact that all of these macrophage populations share numerous myeloid- and macrophage-specific markers, such as ionized calcium binding adaptor molecule 1 (Iba1), F4/80 (mouse) (or EMR1 (human)), and CX3CR1, microglia have their unique signatures. Transcriptome analyses comparing microglia, myeloid, and other immune cells have identified 239 genes and 8 microRNAs that are highly expressed and unique to microglia. These molecular signatures include *Sall1*, *Tgfbr1*, *P2ry12*, *Fcrls*, and *Gpr34* genes that are dependent on the transforming growth factor-*β* (TGF*β*) signaling—an essential pathway required for the development of microglia [16]. Moreover, the same analyses have identified the purinergic receptor P2y12 (P2ry12) as a specific marker for microglia [16]. In addition to the varying markers among different macrophage subsets, CNS-associated myeloid populations also have distinct ontogenesis. Current view supports that microglia originate exclusively from yolk sac-derived hematopoietic progenitors, whereas the other CNS resident macrophage subsets arise later during embryonic development [10, 11, 17]. This view is supported by a series of elegant genetic fate-mapping and parabiosis studies. By injection of tamoxifen into pregnant mice between E7 and E8.5, when embryonic hematopoiesis is limited to the yolk sac, to induce Cre recombinase activity from the runt-related transcription factor 1 (*Runx1*) locus [10] or from the colony-stimulating factor 1 receptor (*Csf1r*) locus, these fate-mapping experiments have demonstrated that the majority of adult microglia are derived from the yolk sac [11]. A similar pattern of microglial cell development also occurs in humans [18]. Parabiosis experiments have also recently shown that the other CNS macrophage subsets, except choroid plexus macrophages, arise from hematopoietic precursors later during embryonic development and become stable populations [19]. Due to the blood-brain barrier, circulating leukocytes (e.g., monocytes, T, B, and natural killer (NK) cells) normally stay within the blood vessels and do not enter the healthy brain, unless the blood-brain barrier is disrupted during CNS diseases, including inflammation, autoimmunity, and cancer. The CNS-infiltrating monocytes give rise to disease-related macrophages and execute distinct functions that differ from resident microglia [20], which we will discuss in Sections 3 and 4.

The development of microglia is controlled by many molecular elements including transcription factors, growth factors, chemokines, microRNAs, and others [21]. One of the important factors that control the microglia population are the signals emanating from the binding of colony-stimulating factor 1 (CSF1) and interleukin 34 (IL-34) to the microglial CSF1 receptor (CSF1R). Mice deficient in the CSF1R or IL-34 or the CSF1R adaptor protein DNAX activation protein of 12 kDa (DAP12) contain substantially reduced numbers of tissue macrophages, including microglia [22, 23]. The transcription factor interferon regulatory factor (IRF)-8 is also essential for the development of microglia, as IRF8-deficient mice show a significantly reduced microglia density in adults [17]. Once the CNS is fully developed, the population size of microglia is maintained via a balance between mitosis and apoptosis [24]. In contrast, the generation of other CNS macrophages relies on the transcription factor PU.1, but not MYB, BATF3, and NR4A1 [19]. A more complete understanding of molecular circuits that regulate the development and homeostasis of CNS microglia and macrophages may lead to improved strategies for better modulating the size of these cellular populations.

### 2.2. Physiological Functions of CNS Microglia and Macrophages

Generic effector functions of macrophages include activities associated with their highly developed lysosomal compartment that bears critical protease and bactericidal activity [25]. Microglia and macrophages are phagocytic cells that constitutively express several families of receptors that facilitate the removal of aged, necrotic tissues, and toxic molecules from the circulation and their surroundings [5, 8]. These receptors include scavenger receptors (e.g., CD36, SR1, and macrophage receptor with collagenous structure (MARCO)), low-density lipoprotein (LDL) receptor family members (e.g., LDLR, ApoER2, and VLDL), and three receptor tyrosine kinases ( Tyro3, Axl, and Mertk) [5, 26]. Mertk and Axl are expressed in resting and activated macrophages, respectively [5]. Engagement of Tyro3, Axl, and Mertk by binding to soluble proteins, growth arrest-specific 6 (GAS6) and protein-S, results in opsonization of apoptotic cells [5, 21]. Macrophages also capture and endocytose immune complexes and complement-opsonized protein complexes through Fc receptors and complement receptors [5, 8, 21, 25]. In addition, macrophages often express chemokine receptors (e.g., CX3CR1 and CXCR4) and integrins (e.g., CD11b and CD11c), which control the migration and positioning of microglia and macrophages within the CNS and enhance their capacity to phagocytose and eliminate bound target cells [21].

Microglia interact with neurons and constitute important components that support the development of the healthy brain [27]. Disruption of these interactions can have a severe negative impact on the functioning of the CNS. Here, we summarize several vital microglia-mediated homeostatic functions that help establish and maintain the overall health of the nervous system, including regulation of neuronal survival and death as well as synaptogenesis. During embryonic development, microglia and perivascular macrophages are uniquely positioned through the pial surface and migrate along the abluminal surface of penetrating vessels to influence the early sprouting, migration, anastomosis, and refinement of the growing CNS vasculature [10]. Microglia also produce various neurotrophic factors that promote the differentiation and survival of neurons. For example, insulin-like growth factor 1 (IGF-1) is released by surrounding microglia to promote the survival of layer V cortical neurons during postnatal development [28]. In adulthood, IGF-1 induces multipotent rat hippocampus-derived neural progenitor cells to differentiate into oligodendrocytes [29]. IGF-1 can also protect immature oligodendrocytes from glutamate-mediated apoptosis [30]. In addition to IGF-1, microglia also secrete other trophic factors, such as basic fibroblast growth factors (FGF), hepatocyte growth factors (HGF), platelet-derived growth factors (PDGF), epidermal growth factor (EGF), nerve growth factor (NGF), and brain-derived neurotrophic factor (BDNF). All of these factors play significant roles in neuronal development, maintenance, and function throughout life [31]. Microglia not only support neuronal survival, but also function as a scavenger to eliminate immature faulty neurons resulting from defective differentiation and/or migration [32]. Microglia induce such neuronal death through the release of soluble factors, such as NGF and reactive oxygen species (ROS) [33, 34].

In addition, microglia play a crucial role in shaping and maintaining the neuronal synaptic network, which occurs constantly throughout life [35]. This type of microglia-mediated remodeling of synapses, called synaptic pruning, is a process in that damaged or unnecessary synapses are eliminated in order for the developing neurons to establish the mature CNS circuit and maintain synaptic homeostasis [35, 36]. The synaptic pruning occurs when an “eat me signal” is created by the engagement of microglial receptor CR3 by the complement protein C3 [37]. In addition to synaptic pruning, microglia also produce various trophic factors and synaptogenic signals to properly regulate synaptic function and plasticity [38]. As a result, reduced microglia in the brain may result in aberrantly increased synaptic activity and a delay in synaptic pruning, leading to cognitive impairments [36, 39]. Finally, the release of neurotransmitters and neuropeptides by neurons promotes neuron-glia communications that fine-tune the homeostatic regulation by microglia [40, 41]. Taken together, the establishment and maintenance of a healthy nervous system requires a tight control of microglia function.

### 2.3. Pathological Function of CNS Microglia and Macrophages

Microglia and macrophages normally function independently of activating stimuli. However, to meet with greater demand for the control of infection or tissue injury, the functional activity of microglia and macrophages can be increased by a variety of stimuli. The nature of these stimuli often determines the distinct morphology and movement of activated microglia to better cooperate their function, as reviewed by others [42–44]. Although this enhanced function allows microglia and macrophages to become more responsive to changes in their surroundings, it also bears the inherent risk of hyperactivation and the ensuing collateral tissue damage. To counterbalance the activatory program, microglia and macrophages are subjected to silencing programs that set tissue-specific thresholds for their activation and allow them to gradually respond to and gauge the quality and intensity of the stimulus [8]. The intensity and duration of this activation or inhibition are balanced through the activating or inhibitory receptors they express. For example, the immunoglobulin superfamily (Ig-SF) molecules deliver either activating or inhibitory signals through protein tyrosine kinase and protein tyrosine phosphatase pathways, respectively. The triggering receptor expressed on myeloid cells 2 (TREM2) is an activating receptor that binds to phospholipids [45], while binding TGF*β* receptor (TGF*β*R), CD33, CD200R1, and signal regulatory protein *α* (SIRP*α*) to TGF*β*, sialic acids, CD200, and CD47 delivers inhibitory signals, respectively [32]. However, less is understood about the roles of tumor necrosis factor (TNF) receptor (TNFR) family members and signaling lymphocytic activation molecule (SLAM) family members in the regulation of macrophage activity [46]. Thus, the imbalance between the activating and inhibitory signals that regulate the activity of microglia and macrophages may pertain to the occurrence of tissue pathology, including both CNS autoimmunity and tumor.

Plasticity and diversity are hallmarks of cells in the macrophage lineage. In response to different stimuli, microglia and macrophages undergo either classical (M1) or alternative (M2) activation [47]. This type of polarized activation of macrophages is often controlled by intrinsic (e.g., epigenetic program) or extrinsic (e.g., inflammatory cytokines) regulatory factors [48]. The M1/M2 continuum has been applied to CNS infiltrating macrophage/monocytes in the context of inflammation or tumor. M1 activation is a proinflammatory and neurotoxic state typically induced by simultaneous triggering of toll-like receptors (TLRs) and interferon (IFN)-*γ* signaling pathways, which is generally associated with immunity to bacteria and intracellular pathogens. These M1 macrophages produce proinflammatory cytokines and chemokines, such as TNF-*α*, interleukin (IL)-6, IL-1*β*, IL-12, and C-C chemokine ligand 2 (CCL2) [47]. M1 macrophages also express the nicotinamide adenine dinucleotide phosphate (NADPH) oxidase, which in turn generates superoxide and ROS, as well as inducible nitric oxidase that converts arginase into nitric oxide (NO) [49]. NO increases the toxic effect of glutamate, thereby potentiating N-methyl-d-aspartate (NMDA) receptor-mediated neurotoxicity [47, 49]. Another important inflammatory mediator produced by M1 macrophage is matrix metalloproteinase (MMP)-12 [47]. Lastly, M1 macrophages often express high amounts of MHC class I or II, costimulatory molecules, Fc receptors, and integrins, which also facilitate induction of inflammation and neurotoxicity [49].

M2 activation describes the anti-inflammatory and tissue remodeling activities of macrophages, which are usually observed in settings dominated by type 2 responses, such as helminth immunity, asthma, and allergy [48]. It can be induced by IL-4, IL-10, IL-13, ligation of Fc receptors by immunocomplexes, and detection of apoptotic cells. Moreover, activation of the transcription factors peroxisome proliferator-activated receptor gamma (PPAR*γ*), liver X receptor (LXR), and retinoic acid receptor (RXR) by fatty acids, oxysterols, and 9-cis-retinoic acid can also trigger the M2 activation state [47]. M2 activation promotes the release of prosurvival factor progranulin [50, 51] and anti-inflammatory cytokines, such as IL-10 and TGF*β*, and induces arginase 1, which promotes the conversion of arginine into polyamines [47, 49]. M2 macrophages secrete growth factors such as IGF-I, FGF, and CSF1, as well as neurotrophic factors such as NGF, BDNF, neurotrophin 4/5, and glial cell-derived neurotrophic factor (GDNF). In turn, these neurotrophic factors engage a family of receptor tyrosine kinases known as tropomyosin-receptor-kinase (Trk) receptors, which regulate synaptic strength and plasticity [27].

Although the M1 and M2 categories have been helpful for conceptualizing macrophage activities *in vitro*, it is increasingly accepted that the M1/M2 paradigm is inadequate to describe microglia and macrophage activation *in vivo*, as they rarely display a significant bias toward either the M1 or M2 phenotype. Indeed, a recent study based on single-cell transcriptome analysis has described a novel microglial cell type associated with neurodegenerative diseases, called disease-associated macrophage (DAM). The genetic programming of this microglial subset involves downregulation of microglial inhibitory-checkpoint pathways in a TREM2-independent manner and subsequent activation of the TREM2-dependent program [52]. This new microglial cell has the potential to restrict neurodegeneration. Another recent study has also identified a type of microglial cell from models of amyotrophic lateral sclerosis (ALS), multiple sclerosis (MS), and Alzheimer's disease (AD) and from tissues surrounding neuritic *β*-amyloid (A*β*)-plaques in the brains of people with AD. This microglial cell carries a specific apolipoprotein E- (APOE-) dependent molecular signature that depends on TREM2-induced APOE signaling pathway, which switches the microglia from a homeostatic to a neurodegenerative phenotype after phagocytosing apoptotic neurons. Targeting the TREM2-APOE pathway has prevented neurodegeneration by restoring the homeostatic signature of microglia [53]. These findings suggest that microglia may have a disease-associated signature common to many CNS disorders, including neurodegenerative diseases, autoimmunity, and possibly cancer, which is worth further investigation.

## 3. CNS Microglia and Macrophages in Autoimmunity: Multiple Sclerosis

CNS microglia and macrophages play important roles in communication between the systemic immune system and the brain. These cells not only regulate the innate immune responses to mediate host defense against cellular or pathogenic components [32, 54], but also modulate the adaptive immune components functioning as antigen-presenting cells [55, 56], or accessory helper cells [32]. Here, we summarize the roles of CNS microglia and macrophages in the regulation of both aspects of immune responses and discuss the contribution of these dysregulated responses to the pathogenesis of CNS disorders, exemplified by multiple sclerosis here.

### 3.1. Microglia and Macrophages in Innate Immunity

As a component of innate immunity, macrophages are critical players in the first line of defenses against infection or tissue injury. This is largely attributed to the vast array of receptors expressed on macrophages. These receptors include pattern recognition receptors (PRRs) that detect pathogen-associated molecular patterns (PAMPs) or tissue damage-associated molecular patterns (DAMPs). PRRs include TLRs (e.g., TLR4 and TLR1/2) and their coreceptors, such as CD14, nucleotide-binding oligomerization domain (NOD)-like receptors (NLRs), receptors for nucleic acids, retinoic acid-inducible gene I (RIG-I)-like receptors, and C-type lectin receptors (CLRs) (e.g., CLEC7A) [57]. Microglia and macrophages also express the receptors for proinflammatory and anti-inflammatory cytokines, such as IFN*α*/*β*, IFN*γ*, TNF*α*, IL-1*β*, IL-10, and TGF*β*, to regulate the intensity of the inflammatory responses [57].

Although the repertoire of these receptors varies among different tissue macrophages and likely reflects local adaptation, these receptors all have important roles in the induction of innate immune responses. They enable microglia and macrophages to engulf and destroy foreign particles and dying cells to promote an M1-like phenotype [57]. Engagement of these receptors also connects to the adaptor myeloid differentiation primary response gene 88 (MyD88). This relays activating signals to regulate inflammasome formation and induce the production of cytokines (e.g., TNF*α* and IL-1*β*), which can further enhance the functional activity of M1 cells [58]. Microglia and macrophages with the M2 phenotype are intimately involved in CNS repair and regeneration, as demonstrated by the role of growth factors, cytokines, and chemokines released by these cells in response to the CNS injury [59, 60]. Microglia and macrophages also secrete MMPs that regulate the deposition of extracellular matrix components at the injured sites [60]. In addition, the protective role of M2 cells may reflect their expression of the IL-4 receptor. Engagement of this cytokine receptor by IL-4 prevents a proinflammatory skew of microglia and macrophages, which influences normal neuronal function and behavior [61, 62]. Finally, microglia and macrophages play a critical role in orchestrating the inflammatory response by provision of chemokines and cytokines, which recruit and activate neutrophils, monocytes, and lymphocytes, to intensify the inflammation [57].

### 3.2. Microglia and Macrophages in Adaptive Immunity

Besides functioning as sentinels, macrophages also function as antigen-presenting cells and participate in the activation of the adaptive arm of the immune response [5]. Upon immunological insults, other innate immune cells, such as NK cells, often provide the initial source of IFN*γ* that enables macrophages to develop a classical activated M1 phenotype [63]. These M1 macrophages then produce large amounts of TNF*α*, IL-12, and IL-23, which are important drivers of type 1 helper T (T_H_1) cell and type 17 helper T (T_H_17) cell responses [64]. T cell-derived IFN*γ* provides a positive amplification feedback loop that expands the M1 cells while increasing their microbicidal and tumoricidal activities [63]. M1 cells are generally believed to display antitumor properties by antagonizing the suppressive activities of tumor-associated macrophages (TAM), myeloid-derived suppressor cells (MDSC), and alternatively activated macrophages and regulatory macrophages, which in turn promote tumor growth, invasion, and metastasis by suppressing adaptive antitumor immune responses [65] (see Section 4). Because M1 macrophages secrete large amounts of TNF*α* and IL-1*β* that contribute to the differentiation of T_H_17 cells, they are also believed to be important drivers of chronic inflammatory and autoimmune diseases, including multiple sclerosis, rheumatoid arthritis, atherosclerosis, pulmonary fibrosis, and Crohn's disease [66–68] (see Section 3.3).

In contrast to M1 cells, M2 cells mainly display suppressive or immunoregulatory activity. They antagonize M1 responses, dampen inflammation, suppress antitumor immunity, and promote wound healing, tissue remodeling, and angiogenesis [5, 69]. Regulatory macrophages that secrete IL-10 have similar roles in adaptive immune responses, although they are particularly adept at suppressing antimicrobial immunity [63]. Regulatory macrophages also facilitate the maintenance of immune homeostasis in the gut by inducing the development of regulatory T cells (Treg) [70], whereas M2 cells mediate secondary immunity to gastrointestinal worms [71]. Although alternatively activated macrophages are induced by a variety of innate IL-4- and IL-13-producing cells, including basophils [72], T_H_2 cells are thought to serve as the main inducers of M2 cells when the adaptive immune response is activated, as in many chronic inflammatory and fibrotic diseases [73, 74]. In the CNS, IL-4 produced by T cells in the meninges and cerebrospinal fluid prevents local inflammation, possibly benefiting cognition through regulation of M2 cells [75].

### 3.3. CNS Microglia and Macrophages in Multiple Sclerosis

**Figure 1:**
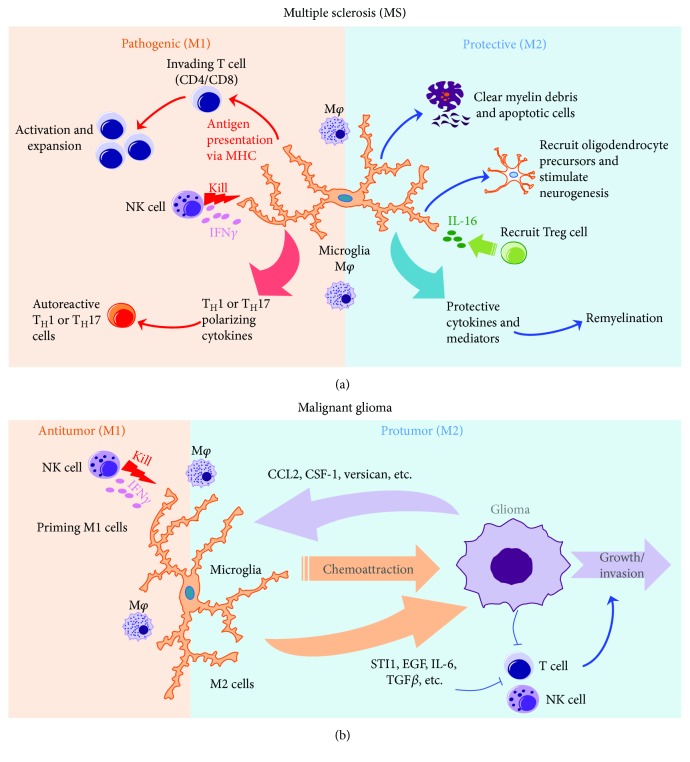
Immune regulation of MS and malignant gliomas by CNS microglia and macrophages. (a) CNS microglia and macrophages (M1) activate autoreactive T cells and program encephalitogenic T_H_1 and T_H_17 cells to induce and exacerbate MS, while NK cells reduce numbers of M1 cells, and M2 cells recruit Treg, contributing to disease amelioration. Microglia also provide protective roles by helping remyelination and neurogenesis. (b) NK cells may prime M1 macrophage and microglia or reduce numbers of M2 cells to promote antitumor response. M2 cells are regulated by factors derived from glioma and further produce suppressive factors to intensify the immunosuppressive environment within the glioma, contributing to the tumor growth and invasiveness.

As discussed in Section 2.3, M1 macrophages may contribute to many autoimmune diseases. Here, we focus on multiple sclerosis (MS) (Figure 1). MS is a CNS disease that affects over 2 million people and has no known cure. MS is considered as a chronic autoimmune inflammatory disease affecting brain, nerve, and spinal cord tissues, which causes demyelination of neurons, axonal damage, and neurodegeneration [76]. Myelin-specific T_H_17 and T_H_1 cells and B cells are believed to help initiate and/or promote the development of MS [76]. Experimental autoimmune encephalomyelitis (EAE) is the most commonly used animal model for MS and is induced by CD4^+^ T cells specific for myelin-derived antigens, either generated after immunization or injected directly [77]. Studies using this EAE model have shown that microglia and macrophages contribute to aggravating the CNS pathology [78]. In mice with the deletion and/or inactivation of microglia, delayed EAE onset and reduced severity of clinical symptoms are observed along with decreased inflammation, confirming the crucial role of microglia in the pathogenesis of MS [78].

Microglia contribute to EAE disease initiation by presenting antigens to naive T cells and secreting cytokines, such as IL-6, IL-23, IL-1*β*, and TGF*β*, that are required for the differentiation and activation of encephalitogenic T_H_17 cells. It remains unclear if microglia and macrophages regulate the other T_H_ cells that modulate EAE and MS progression. It is known, however, that activation or inhibition of effector T cells by microglia is controlled by other neighboring immune cells. For example, a subset of microglia has the capacity to suppress effector T cell proliferation by inducing FoxP3^+^ Treg, leading to attenuation of EAE disease progression [79]. Although it is believed that microglia have a neurotoxic role in MS and EAE, there is conflicting evidence that suggests microglia exert a neuroprotective function in MS and EAE [42]. Potential beneficial effects of microglia in EAE and MS are thought to occur in at least three major ways: (1) microglia clear myelin debris and apoptotic cells; (2) microglia release protective cytokines and mediators for remyelination; and (3) microglia trigger recruitment of oligodendrocyte precursors and stimulate neurogenesis [27, 32]. The neurotoxic and neuroprotective functions of microglia may depend on the CNS disease stages and activation status of microglia, which awaits further investigation. Interestingly, a recent study using the parabiosis model combined with highly efficient permanent labeling of blood monocytes has elegantly revealed that circulating monocytes invade the inflamed CNS during EAE pathogenesis and have an essential role in promoting disease progression [80]. A precise understanding of these two pools of CNS macrophage subsets during CNS inflammation and autoimmunity may provide insights into better strategies for the treatment of these disorders.

## 4. CNS Microglia and Macrophages in Cancer: Malignant Glioma

As discussed in Section 2.3, M1 macrophages display antitumor activity while M2 cells are protumorigenic. Here, we discuss one of the most deadly brain cancers, malignant glioma (Figure 1). Gliomas, a type of brain tumor that grows from glial cells, include astrocytoma, oligodendroglioma, and glioblastoma. Gliomas are complex tumors composed of both neoplastic and nonneoplastic cells. The majority of nonneoplastic cells are TAMs, which account for 50% of the cellular fraction of gliomas. TAMs include infiltrated monocyte-derived macrophages and brain-resident microglia. These cells constitute a supportive stroma for neoplastic cell expansion and invasion [81]. Therefore, understanding the cellular and molecular mechanisms for the regulation of microglia and macrophages may suggest novel strategies to target these cells for immunotherapy of gliomas.

The importance of microglia and macrophages in glioma is underscored by clinical observations. The number of infiltrated TAMs and microglia, identified by CD68 and Iba-1 antibodies, respectively, is positively correlated with tumor grade [82] and inversely correlated with the recurrence-free survival of patients [83]. While monocytes represent 10–15% of the cell population in normal nonneoplastic brain specimens, 15–30% of cells in low-grade gliomas are TAMs [84]. Moreover, the proportion of microglia can reach 35–50% within the gliomas, depending on the region in which the tumor arises and the degree of tumor invasiveness [85]. Microarray analyses have revealed approximately 1000 transcripts that are highly enriched in glioma-associated microglia and macrophages relative to control microglia. Interestingly, these genes show little overlap with reported gene signatures for M1 or M2 phenotypes [86].

Despite the positive correlation between the number of intratumoral TAM and microglia with glioma malignancy, it remains controversial and to be determined whether these cells display antitumor activity or protumorigenic properties. Understanding these mechanisms is important for directing future therapeutic strategies for glioma. Deletion of microglia and macrophages increases glioma tumor volume by 33%, suggesting that these cells may contribute to the antitumor response [87]. In contrast, pharmacological activation of microglia and macrophages results in increased glioma size, indicating that these cells may promote tumor growth and invasion [88]. Moreover, in the presence of microglia, the motility of the murine glioma cells is increased threefold *in vitro* [89]. Using transgenic mice expressing the herpes simplex virus thymidine kinase gene under the control of the *Cd11b* promoter, Galarneau et al. have shown that targeted reduction of CD11b^+^ microglia and macrophages concomitantly results in attenuated glioma growth *in vivo* [44, 90]. Within the tumor microenvironment, the crosstalk between glioma cells and microglia/macrophages may determine the glioma aggressiveness and invasiveness. Microglia release several factors to promote glioma proliferation and/or migration. Microglia synthesize and release stress-inducible protein 1 (STI1), a cellular prion protein ligand that increases the proliferation and migration of glioblastomas *in vitro* and *in vivo* [91], as well as EGF, which stimulates glioblastoma cell invasion [92]. TGF*β*, predominantly released from microglia, also increases the migration of glioma cells; moreover, blocking TGF*β* signaling impairs glioma growth [93]. In addition, TGF*β*2 induces the expression of MMP2 in glioma cells and suppresses the expression of tissue inhibitor of metalloproteinases (TIMP)-2, which degrades the extracellular matrix and subsequently promotes glioma invasion [94]. TAMs not only target glioma cells, but also indirectly affect tumor growth through angiogenesis. This likely occurs via expression of the receptor for advanced glycation end product (RAGE) and vascular endothelial growth factor (VEGF), an important proangiogenic factor [95].

On the other hand, factors produced from glioma cells facilitate the glioma-promoting activity of microglia. CSF1, constitutively released by the glioma cells, acts as a chemoattractant for microglia and also converts microglia into a protumorigenic phenotype [96]. CCL2 is another factor released from glioma cell lines and acts on the CCL2 receptor (CCR2) expressed on microglia [97]. CCL2 can trigger the release of IL-6 from microglia, promoting the glioma invasiveness [98]. Glioma-derived versican interacts with TLR2, inducing CNS microglia and macrophages to express membrane type 1-matrix metalloproteinase 1 (MT1-MMP) that activates MMP2 [99]. In its active form, MMP2 amplifies the glioma-brain macrophage interaction network and potentiates glioma growth and invasiveness [99]. Furthermore, the suppressive factors produced from both glioma and microglia or TAMs inhibit the antitumor activity of effector CD4^+^ and CD8^+^ T cells and NK cells, but promote the recruitment and suppressive activity of Treg and MDSC, which constitute the immunosuppressive microenvironment and enhance glioma growth [100].

Glioblastomas contain a subpopulation of cells with stem cell-like properties, called glioma stem cells (GSCs), which have the capacity for self-renewal, the potential for multilineage differentiation, and are capable of reconstituting the native tumor following implantation into naive hosts [101]. However, these GSCs reside in the perivascular niche and are highly resistant to radiation and chemotherapy [101]. There is a positive correlation between the density of GSCs and TAMs, indicating that GSCs may recruit TAMs more efficiently than their more differentiated neoplastic counterparts [102]. GSCs also release periostin, which acts as a chemoattractant for TAMs through interactions with TAM's integrin receptor *α*_v_*β*_3_ [103]. TAMs also influence the properties of GSCs, in that TGF*β* released from TAMs induces MMP-9 expression and increases GSC invasiveness [104]. In addition, naive microglia can reduce the sphere-forming ability of human stem cells and in turn, suppress glioma growth. In contrast, microglia or TAMs cultured from glioma patients lack this antitumorigenic potential [105]. It is likely that GSCs secrete factors, which inhibit the phagocytosis activity of TAMs and induce the secretion of cytokines to prevent antitumor responses [106].

Due to the importance of microglia and TAMs in glioma growth and invasiveness, these cells are currently considered as therapeutic targets. Interfering with CSF1 signaling by antibody-mediated blockade or use of CSF1R inhibitors is a potential approach to regulate glioma growth by targeting TAMs [96]. Periostin has also emerged as an interesting target for attenuating the tumor-supportive phenotype of TAMs by interrupting integrin *α*_v_*β*_3_ signaling [103]. Interfering with this pathway via a blocking peptide impairs TAM recruitment. Finally, Minocycline, an antibiotic that interferes with the process of microglia activation and has the unknown effects on tumor growth, is currently being tested in a clinical trial of MS patients [107]. However, as discussed above, the dual antitumoral and protumoral activities of microglia and macrophages should be taken into account when the therapeutic strategy for malignant glioma is configured. Additionally, therapeutic strategies should evaluate the crosstalk of microglia and macrophages with other immune cells, as reviewed below.

## 5. Regulation of CNS Disorders: Crosstalk between Macrophage and NK Cells

We have discussed the highly diverse and complex function of microglia and macrophages during CNS autoimmunity—multiple sclerosis and cancer—malignant glioma. Considering the important roles of innate immune components in host defenses against these two types of CNS disorders, here we emphasize the crosstalk between CNS microglia/macrophages and NK cells, one of the important components of innate immunity, which has not been reviewed elsewhere. We focus on the discussion of how this type of cellular interactions impact on the pathological processes of both CNS disorders.

Macrophages regulate the functional activity of various innate immune subsets, including neutrophils, innate lymphocyte cells, and NK cells. NK cells exhibit potent cytotoxicity and produce cytokines in response to inflammation and stressed conditions, contributing to many facets of immune surveillance and tolerance [108]. It is well-recognized that the macrophage-NK interaction is a major first-line defense against pathogens. However, the crosstalk between macrophages, particularly microglia, and NK cells in the regulation of tissue-specific immune responses remains largely unknown.

Macrophages can activate or inhibit NK cell activity through either direct cell-to-cell contact via a diverse receptor-ligand interaction or soluble mediators, such as cytokines [109]. Conversely, NK cells also regulate the population size and functional activity of macrophages [109]. The outcome of the macrophage-NK interaction depends on the tissue origin of macrophages [110]. Interestingly, macrophages derived from peripheral blood mononuclear cell (PBMC) do not display the similar regulatory property as tissue-resident macrophages. The intensity and duration of macrophage-NK crosstalk depend on the nature of stimuli. For example, high doses of lipopolysaccharide (LPS) induce the expression of various ligands of the activating receptor NKG2D in human macrophages, UL16-binding proteins (ULBP1, ULBP2, and ULBP3) and MHC class I-related chain A (MICA) [111]. Human NK cells that are in contact with LPS-activated macrophages express increased levels of NKG2D. Consequently, NK cells lyse these macrophages stimulated with high doses of LPS to prevent endotoxic shock [111]. In contrast, LPS-stimulated microglia are less susceptible to NK cell-mediated cytotoxicity compared to resting microglia, likely due to reduced NKG2D expression in NK cells upon interactions with LPS-stimulated microglia [111]. Subsequently, this may help microglia present antigens to infiltrating T cells and initiate the immune response in the brain [112]. Other receptor-ligand pairs, including 2B4-CD48, NKp46-NKp46 ligand, CD226-CD112/CD155, and NKp80-AICL, also induce similar crosstalk effects as the NKG2D-NKG2D ligand on macrophage-NK cells, but only NKp46 engagement has been implicated in the NK-mediated killing of microglia [112]. Besides increased NK cytotoxicity, activated macrophages may also induce the release of IFN*γ* by NK cells that further amplifies the ongoing immune responses. In addition to activating interactions between NK cells and macrophages, there is also inhibitory crosstalk. We and others have previously reported that Qa-1, the homologue of human HLA-E and a ligand for the NK cell inhibitory receptor NKG2A, is upregulated on the surface of activated macrophages. Despite the unaltered NKG2A expression, the NKG2A-Qa-1 interactions allow the macrophages to escape NK cell-mediated lysis [113–115]. Consequently, blockade of the interaction between NKG2A on NK cells and Qa-1 on microglia by an anti-NKG2A antibody unleashes NK cell activity, reduces microglia activation, and decreases T cell infiltration into the CNS, leading to amelioration of EAE [114]. Due to the enhanced NK cell activity via the anti-NKG2A-mediated blockade, this antibody has also been applied in the clinical trials of multiple cancers (e.g., NCT02331875 and NCT02557516). It will be worthy to test the therapeutic efficacy of anti-NKG2A as a new generation of checkpoint inhibitors in the treatment of malignant glioma. Besides direct contact between macrophages and NK cells, their crosstalk is also regulated by the cytokines they produce. Macrophages produce IL-12, IL-15, IL-18, and IL-23 to induce the production of IFN*γ*, TNF*α*, or granzyme B by NK cells [109], whereas TGF*β*1 and IL-10 released by macrophages, especially TAMs, inhibit NK cell function [109]. The latter may contribute to the exhausted or dysfunctional phenotype of NK cells, as observed in many tumors, including malignant glioma [100], which promotes tumor growth and invasion. Given the dynamic interaction between microglia/macrophages and NK cells that regulates the CNS inflammation, autoimmunity, and tumor, a more complete understanding of their molecular interplay may guide the development of optimal interventions of these CNS disorders.

## 6. Conclusion

As a resident macrophage population, microglia are critical components in the establishment and maintenance of a healthy nervous system. They not only purge damaged or unnecessary neurons and synapses, but also act as the primary form of active immune defense against infectious and stress-derived agents. Microglia and the CNS-infiltrating monocyte-derived macrophages actively participate in the regulation of innate and adaptive immune responses under pathological insults. We have discussed two types of CNS disorders here, multiple sclerosis with excessive immune responses and glioma with extreme immunosuppression. Although the cellular components share similarities between these two types of diseases (Figure 1), the mechanistic actions of CNS microglia and macrophages and their interactions with other immune cells are fully context-dependent. Additional studies are needed to dissect the differential contribution of microglia versus CNS-infiltrating monocyte-derived macrophages to these disorders. The discovery of P2ry12 as a specific marker for microglia definitely facilitates a more precise understanding of these macrophage populations. In the future, a better understanding of molecular circuits that regulate the homeostasis and function of these macrophage populations may also direct more effective therapeutic strategies that specifically target individual subsets for better therapy of CNS autoimmunity, cancer, and other neurodegenerative disorders.
